# Influence of GdVO_4_:Eu^3+^ Nanocrystals on Growth, Germination, Root Cell Viability and Oxidative Stress of Wheat (*Triticum aestivum* L.) Seedlings

**DOI:** 10.3390/plants10061187

**Published:** 2021-06-10

**Authors:** Anna Ekner-Grzyb, Jagna Chmielowska-Bąk, Agata Szczeszak

**Affiliations:** 1 Department of Plant Ecophysiology, Faculty of Biology, Institute of Experimental Biology, Adam Mickiewicz University, Uniwersytetu Poznańskiego 6, 61-614 Poznań, Poland; jagna.chmielowska@amu.edu.pl; 2 Department of Rare Earths, Faculty of Chemistry, Adam Mickiewicz University, Uniwersytetu Poznańskiego 8, 61-614 Poznań, Poland; agata_is@amu.edu.pl

**Keywords:** nanotoxicology, lanthanide-doped nanoparticles, vascular plants, phytotoxicity, lipid peroxidation, cell viability

## Abstract

The increasing application of lanthanide-doped nanocrystals (LDNCs) entails the risk of a harmful impact on the natural environment. Therefore, in the presented study the influence of gadolinium orthovanadates doped with Eu^3+^ (GdVO_4_:Eu^3^) nanocrystals on wheat (*Triticum aestivum* L.), chosen as a model plant species, was investigated. The seeds were grown in Petri dishes filled with colloids of LDNCs at the concentrations of 0, 10, 50 and 100 µg/mL. The plants’ growth endpoints (number of roots, roots length, roots mass, hypocotyl length and hypocotyl mass) and germination rate were not significantly changed after the exposure to GdVO_4_:Eu^3+^ nanocrystals at all used concentrations. The presence of LDNCs also had no effect on oxidative stress intensity, which was determined on the basis of the amount of lipid peroxidation product (thiobarbituric acid reactive substances; TBARS) in the roots. Similarly, TTC (tetrazolium chloride) assay did not show any differences in cells’ viability. However, root cells of the treated seedlings contained less Evans Blue (EB) when compared to the control. The obtained results, on the one hand, suggest that GdVO_4_:Eu^3+^ nanocrystals are safe for plants in the tested concentrations, while on the other hand they indicate that LDNCs may interfere with the functioning of the root cell membrane.

## 1. Introduction

Recently, a growing interest in the use of nanoparticles (NPs) has been observed. Their unique properties have been utilized in chemical and biological sciences, medicine, as well as in industry and agriculture. However, along with many advantages, their use entails some risks. They can enter the environment and may have impact on the wildlife and human health [1,2,3]. The effects of NPs may be completely different to those of their larger counterparts (bulk materials) [4,5,6,7]. These differences result from the higher surface area to volume ratio and higher surface reactivity of nanostructures compared to the bulk compounds. Moreover, due to their smaller size NPs can enter organisms more easily as compared to bulk structures [4], although this is not always the case [5].

Previous research has shown that NPs are taken up by plants and they are subsequently spread and accumulated in the tissues, mainly in roots, stems and leaves [8,9,10,11]. The NPs influence the contaminated plants in many ways [12,13,14]. They may negatively affect germination, cells morphology, growth rate or final biomass of plants and induce oxidative stress [6,15,16,17,18,19,20,21,22,23]. However, some authors have found no negative consequences or have even observed a biostimulating effect of NPs on plants including *Avena sativa* L., *Zea mays* L., *Lycopersicon esculentum* L., *Lepidium sativum*, *Sinapis alba*, *Sorghum saccharatum*, *Lactuca sativa* L. and *Triticum aestivum* L. [24,25,26,27,28]. The positive effect occurred mainly at low concentrations and was reversed at higher doses of NPs [16,17]. For example, Peng and co-authors showed that NaYF_4_: Yb^3+^, Er^3+^, Tm^3+^ coated with citric acid reduced the growth rate of mung beans at concentrations of 100 µg/mL, whereas a concentration of 10 µg/mL improved the development of the plants [16]. The improvement in growth may be caused by more effective uptake of water or nutrients in plants exposed to NPs [17,26,29], as well as interference with the toxic metals taken up from the medium [30,31]. The overall effect of NPs on plants depends on numerous factors, such as time of the exposure, surface contamination, size, plants species, etc. [32,33,34,35].

Inorganic nanocrystals doped with lanthanide ions (lanthanide-doped nanocrystals, LDNCs) have been widely investigated. Their unique spectroscopic properties, such as their luminescence properties, resistance to photobleaching and photochemical degradation have been found very attractive for many potential applications [36,37]. LDNCs are used in innovative technologies, the optoelectronic industry (e.g., LCD), agriculture, as well as biological and medical sciences (e.g., drug delivery and bioimaging) [38,39,40,41]. Among them, gadolinium-based LDNCs such as vanadates gadolinium doped with Eu^3+^ ions (GdVO_4_:Eu^3^) may be applied as catalysis, polarizers, laser hosts and luminescent materials [42,43]. Moreover, GdVO_4_:Eu^3+^ NPs can be used in biological and medical science, e.g., for magnetic resonance imaging (MRI) and drug delivery [44,45,46,47,48,49].

Nanostructures used in the above-mentioned applications may be transferred to the environment. According to a study in California, the highest amount of gadolinium was observed in the water bodies close to hospitals and medical centers. This discovery suggests that the Gd-based contrast agents used for magnetic resonance imaging are the main source of contamination [50]. Gadolinium may also accumulate in animal tissues, such as the human brain [51]. A study of vertebrate cell lines has revealed that gadolinium may influence the vertebrate cells, e.g., cause apoptosis [52]. Wysokińska et al. have reported that NaGdF_4_:Yb^3+^,Er^3+^, NaGdF_4_ or NaGdF_4_:Eu^3+^ NPs may negatively affect the viability of macrophages and fibroblasts [39]. Our previous study conducted on LDNCs similar to those used in this study shown that Fe_3_O_4_@SiO_2_@GdVO_4_:Eu^3+^ 5% core@shell type nanostructure had no effect on the erythrocyte sedimentation rate, morphology of red blood cells or their membrane permeability when present in concentrations up to 1 mg/mL [53]. However, our other research has shown that the effect of GdVO_4_:Eu^3+^ 5% depends on the surface functionalization, kind of cells and concentration [54].

Few authors have shown interest in the toxicity of LDNCs to plants. According to the literature data, LDNCs may be taken up by particular parts of plants and their presence may influence plant development, similarly to other nanostructures [9,10,16,17,18,55,56]. Moreover, most of the previously published data on the phytotoxicity of LDNCs concerns fluorides, mainly NaYF_4_ [9,10,16,17,57] or oxides [18]. According to the current state of knowledge, no data have been published on the phytotoxicity of vanadates gadolinium doped with europium ions. In the present study, the impact of GdVO_4_:Eu^3+^ NPs on wheat seedlings was investigated.

## 2. Results

### 2.1. Nanoparticles Characterisation and Elemental Analysis

**Figure 1:**
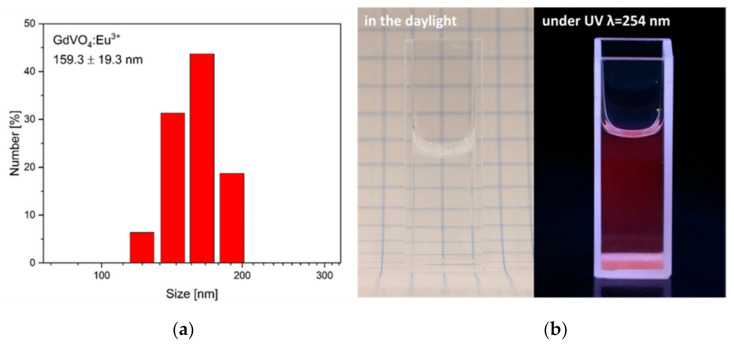
DLS analysis of the GdVO_4_:Eu^3+^ NPs (**a**). Photographs presenting the colloidal GdVO_4_:Eu^3+^ NPs in daylight (**left**) and under 254 nm UV light excitation (**right**) (**b**).

The NPs’ structure and morphology were characterized on the basis of DLS measurements and TEM images. DLS analysis showed that the hydrodynamic diameter of NPs, including their agglomerates, varies between 140 and 180 nm (Figure 1a). Furthermore, TEM image analysis confirmed that NPs form agglomerates with an average grain size of 70 ± 5 nm. Additionally, a negative zeta potential (−20.0 ± 4.29 mV) indicates that NPs colloids are stable at physiological pH and appropriate to use as water colloids [44]. To show the stability and transparency of GdVO_4_:Eu^3+^ NPs aqueous colloids at a concentration of 10 mg/mL, pictures were taken in daylight (Figure 1b, left side). Furthermore, red luminescence under 254 nm UV excitation related to the presence of Eu^3+^ emitting ions was also performed (Figure 1b, right side).

ICP-OES analysis revealed that the LDNCs were taken up by the wheat seedlings.

### 2.2. Growth and Germination of the Seedlings

**Figure 2:**
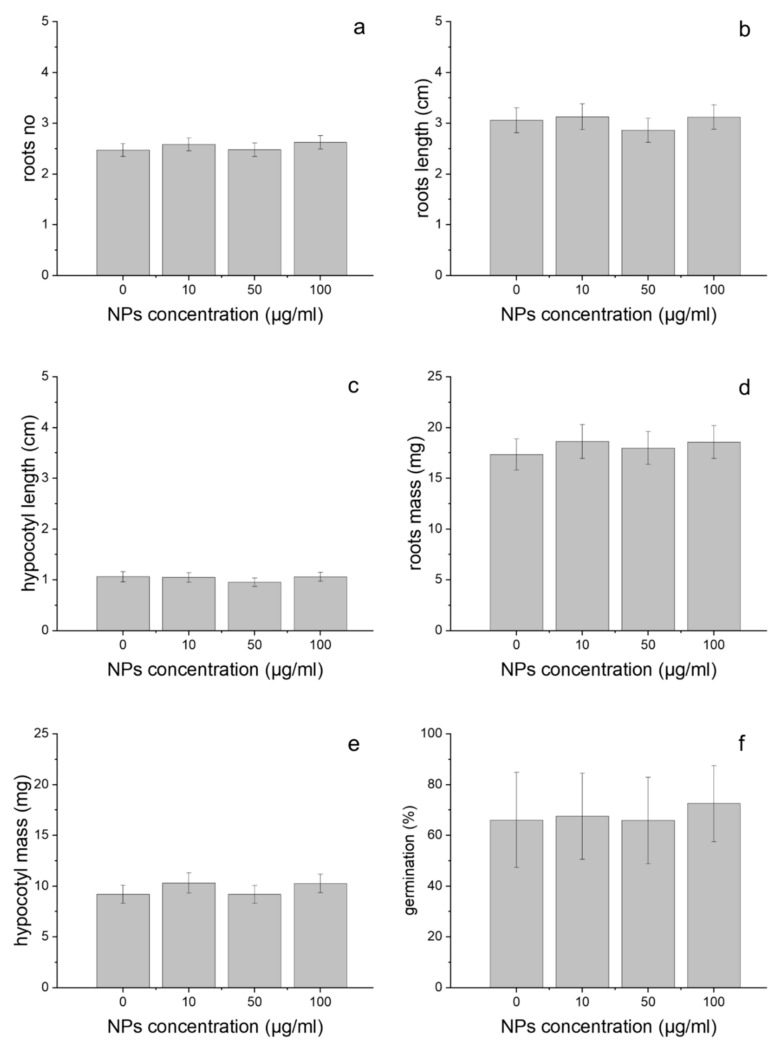
Number of roots (**a**), root length (**b**), hypocotyl length (**c**), root mass (**d**), hypocotyl mass (**e**) and germination rate (**f**) of wheat, treated for 3 days with the GdVO_4_:Eu^3+^ NPs at concentrations: 0, 10, 50 and 100 µg/mL. The results are presented as mean ± SE.

Treatment with GdVO_4_:Eu^3+^ NPs had no significant impact on the morphology of wheat seedlings. The influence of the GdVO_4_:Eu^3+^ NPs on several growth endpoints, including the number of roots, root length, hypocotyl length, root mass and hypocotyl mass was investigated. These parameters were not significantly changed as a result of the plants’ exposure to 10, 50 and 100 µg/mL of LDNCs for 3 days, relative to the controls (Kruskal–Wallis test, *p* > 0.1; Figure 2a–e). The studied LDNCs also did not affect the germination rate of the tested concentrations (Kruskal–Wallis test, *p* > 0.1; Figure 2f).

### 2.3. Oxidative Stress

**Figure 3:**
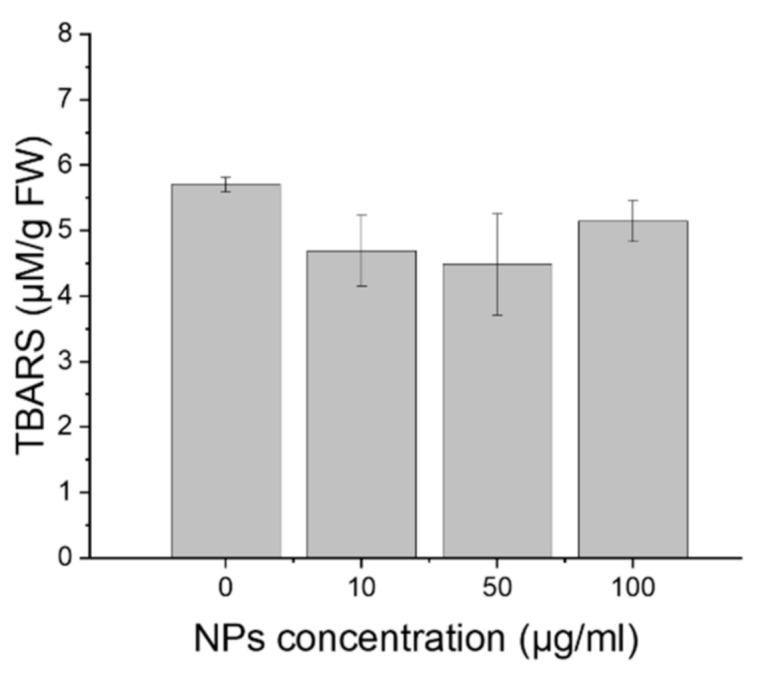
Lipid peroxidation of wheat cells, treated for 3 days with the GdVO_4_:Eu^3+^ NPs at concentrations: 0, 10, 50 and 100 µg/mL. The results are presented as mean ± SE.

The degree of lipid peroxidation, estimated on the basis of the amount of TBARS did not change during the experiments. Treatments of the wheat with GdVO_4_:Eu^3+^ NPs did not affect this factor, at all tested concentrations (ANOVA, *p* = 0.36; Figure 3).

### 2.4. Cell Viability

**Figure 4:**
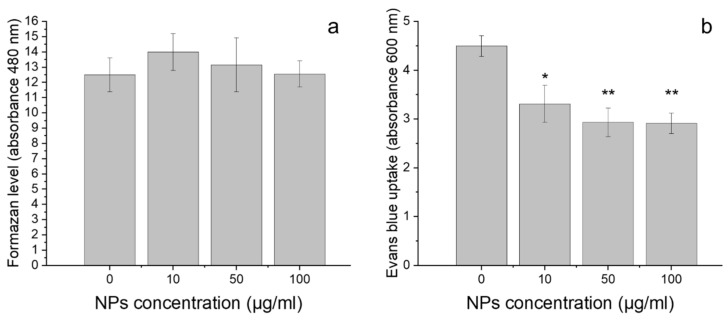
Cell viability of wheat exposed for 3 days to GdVO_4_:Eu^3+^ NPs at concentrations: 0, 10, 50 and 100 µg/mL, determined by TTC (**a**) and Blue Evans (**b**) staining assays. Asterisks (*) indicate statistically significant differences between the samples exposed to NPs and the control, Tukey’s test (* *p* < 0.05 level, ** *p* < 0.01 level). The results are presented as mean ± SE.

The results of TTC staining showed that the exposure of the cells to GdVO_4_:Eu^3+^ NPs had no impact on the amount of formazan level (ANOVA, *p* = 0.83; Figure 4a). However, BE uptake was significantly different in control root cells and the cells incubated with LDNCs (ANOVA, *p* < 0.005). The roots growing in the medium without LDNCs contained significantly more BE dye than those growing in the medium with NPs, both at the 10 µg/mL (Tukey’s test, *p* < 0.05) and the 50 or 100 µg/mL (Tukey’s test, *p* < 0.01) concentrations (Figure 4b).

## 3. Discussion

In the presented study, wheat seeds were incubated with GdVO_4_:Eu^3+^ NPs for 3 days. Even after this short treatment time, the presence of LDNCs was detected inside the plants. However, results of the plants’ growth assays showed that GdVO_4_:Eu^3+^ NPs had no significant effect on germination rate, seedling elongation and biomass of plants, when used in all tested concentrations (Figure 2). According to the current state of knowledge, there are no previous studies on the phytotoxicity of GdVO_4_:Eu^3+^ NPs. Although the LDNPs studied are poorly water soluble [58], some small amounts of the ions can be released to the solutions. Therefore, along with checking the influence of GdVO_4_:Eu^3+^ NPs on wheat plants, we have to consider the effect of their particular compounds (e.g., ions). Similarly to our findings for wheat, Blinova et al. suggested that the oxides containing gadolinium (Ce_0.9_Gd_0.1_O_2_, LaFeO_3_, Gd_0.97_CoO_3_) were not toxic for *Lemna minor* even at 100 µg/mL [59]. According to other authors, gadolinium caused growth inhibition of Chlorophyta [18,29], although Romero-Freire suggest that this effect was observed only when Gd^3+^ ions were mixed with Ce^3+^ and Lu^3+^ lanthanide ions. In another study, Gd_2_O_3_ NPs caused a decrease in roots’ growth of *Triticum aestivum* and *Brassica napus* [60]. This effect depended on the sequence of processes of soaking and incubating during treatments. In the same study, germination of wheat and rape seeds was not affected by the presence of gadolinium oxides NPs [60]. However, Gd^3+^ ions significantly decreased shoot growth of *Zea mays* at 10 µg/mL [61]. Ruíz-Herrera et al. have shown that GdCl_3_ affects the length of primary roots in a dose-dependent manner [62]. Unexpectedly, the same study revealed that with an increasing concentration of gadolinium ions, the total number of lateral roots per plant and lateral roots’ density increased [62]. It has been reported that europium, another lanthanide component of the studied GdVO_4_:Eu^3+^ NPs, may accumulate in macrophytes and alter the plants’ growth [63,64]. The results obtained for one more component of the NPs studied, i.e., vanadium or vanadium oxide, which do not represent lanthanides, have been inconclusive. It has been suggested that plants incubated with a high concentration of vanadium have shorter root length and poorer survival, whereas a low concentration of vanadium may improve the growth rate, accelerate flowering and biomass production [65], see the review in [66].

It has been demonstrated that modulated nutrient content in the tested plants may have an impact on the growth rate. Garcia-Jimenez et al. reported that a solution of NH_4_VO_3_ added at a concentration of 5 mM stimulated the uptake of nutrients by the plants; when the concentration of this compound was increased to 15 mM, the stimulating effect was noticeable but reduced. Exposure to NH_4_VO_3_ at 5 µM has been found to cause an increase in the level of amino acids and sugars in leaves and roots [65]. Other studies have revealed that the presence of europium causes a decrease in the concentration of macroelements in *Lathyrus sativus* roots [63]. In the same paper, it was reported that europium activated proteolytic enzymes, and therefore increased the concentration of amino acids in the cells, causing plants to be less vulnerable to an arid environment [63]. Saatz et al. have demonstrated significant correlations between the concentration of gadolinium in the nutrient solution and the concentration of Ca, Mg and P in the root tissues [61]. However, in the present study, it is unlikely that exposure to GdVO_4_:Eu^3+^ NPs had an impact on seedlings mineral composition as the seedlings were grown in water (or colloids with LDNCs). The differences between the literature data and our results on the growth and development of wheat may be related to several factors, such as differences in the solubility and structures of NPs, as well as the assays’ duration. Moreover, the toxicity may depend on the presence of compounds other that the tested one [30,67].

The effect of NPs on lipid peroxidation [19,67,68,69], see the review in [13], and cell viability [8,32,68,70] has been studied; however, according to the present state of knowledge, the influence of LNDPs on the cell membrane damage and lipid peroxidation in plants has not been studied as yet. However, it has been shown that vanadium ions present at a concentration 50 mg V in 1 kg of soil caused cell death, reduction in the membrane integrity and activation of antioxidant enzymes in chickpea *Cicer arietinum* L. [71]. In our study, the oxidative stress of the seedlings was evaluated by measuring the amount of lipid peroxidation product (thiobarbituric acid reactive substances, TBARS) as a biomarker. The assay showed that the GdVO_4_:Eu^3+^ NPs did not cause oxidation of the membrane lipid of the wheat root cells when used in all tested concentrations (Figure 3).

The effect of the NPs presence on the cells’ viability was assessed by two methods: TTC and EB assays. They provided different results. According to the TTC results, the presence of LDNCs had no impact on the formazan level, when used in all tested concentrations. This suggests that LDNCs did not cause cell death during the treatment. However, the results of the EB assay revealed that incubation of the seedlings with GdVO_4_:Eu^3+^ NPs induced significant alterations in cells’ staining by EB dye. It has been proved that the presence of EB dye in cells is correlated with the loss of integrity in the plasma membrane of the cells and their mortality [72]. Unexpectedly, in the present study, the control cells had higher absorbance than the treated ones (Figure 4b). The mechanism of the observed phenomenon is not known, but several explanations can be proposed. First, the obtained results might be caused by the decreased uptake of EB dye by the cells incubated with the LDNCs compared to the control. The LDNCs may create aggregates, and therefore block pores of the cells, which prevents the access of the dye to the inside of the cells. Indeed, it has been shown that NPs might adhere to cell membranes, and thus, they may alert intracellular transport [73]. Alternatively, the LDNCs may interact with ions or structures that are responsible for pumping EB dye out.

## 4. Materials and Methods

### 4.1. Nanoparticles Characterisation and Elemental Analysis

The NPs used for the ecotoxicity investigation were based on an inorganic matrix of orthovanadates doped with Eu^3+^ ions, GdVO_4_: Eu^3+^, synthesized under hydrothermal conditions. Detailed description of the synthesis method and photophysical characterization of the NPs used are presented in a previous paper [42]. After the synthesis of NPs, the concentration of the stock solution was 3.54 mg/mL as determined by inductively coupled plasma-optical emission spectrometry (Varian ICP-OES VISTA-MPX). Before the assays, transmission electron microscopy (TEM) images were recorded using an HRTEM JEOL ARM 200F transmission electron microscope at the accelerating voltage of 200 kV. Dynamic light scattering (DLS) and zeta potential measurements were performed using a Malvern Zetasizer Nano ZS instrument. The concentration of the colloid sample was 1 mg/mL. Zeta potential was measured at physiological pH.

In order to evaluate the uptake of LDNCs by the plant organisms, elementary analysis was used. After 3 days of incubation, the seedlings were collected, weighed, rinsed, and mineralized in a pressure reactor with HNO_3_. Next, the samples were analyzed using inductively coupled plasma optical emission spectroscopy (ICP OES).

### 4.2. Plant Growth and Treatment

Wheat (*Triticum aestivum* L.) seeds were kindly supplied by the Danko Company (Kościan, Poland). The seeds were surface sterilized for 5 min. with 75% ethanol and another 10 min. with 1% sodium hypochlorite according to [74]. Afterwards, the seeds were washed with tap water for 30 min. and immersed in distilled water for 10 min.

Next, the seeds were incubated with the GdVO_4_:Eu^3+^ NPs according to [32], with some modifications. The seeds were placed in Petri dishes (30 seeds per dish) and filled with colloids of LDNCs at the following concentrations: 0, 10, 50 or 100 µg/mL of the aqueous solution. The concentrations were determined on the basis of the results of previous studies conducted on similar nanoparticles, in which the authors demonstrated that a concentration of 100 µg/mL and lower were enough for optical and MRI imaging [44,47,49,75]. The colloids were sonicated before all assays. They were grown in the dark at a stable temperature of 22 °C for 3 days.

### 4.3. Measurement of Growth Parameters

Germination rate, seedling growth (root and hypocotyl length), number of roots and fresh weight (root and hypocotyl mass) were measured. Six replicates (each consisting of 30 seedlings) were run per treatment.

### 4.4. Cell Viability Estimation

Cell viability was evaluated by two spectrophotometric methods: staining with Evans Blue (EB) and tetrazolium chloride (TTC). The first method is based on the accumulation of EB in cells with damaged membrane. The measurements were performed according to the procedure given in [72] with some modifications. Approximately 200 mg of roots were cut off on the ice and incubated with 0.25% Evans blue (Sigma, E-2129, St. Louis, MO, USA) for 20 min at room temperature. Afterwards, the roots were washed twice for 15 min. with distilled water, homogenized in a mortar with the destaining solution (50 mL of ethanol, 49 mL of distilled water, and 1 mL of 10% SDS) and incubated in the heating block for 15 min at 50 °C. Next, the samples were centrifuged (12,000 rpm, 20 °C, 15 min) and the absorbance of the supernatant was measured at 600 nm. Each experiment was repeated four times and all samples in each experiment were tested in duplicate.

The second method consisted of the measurement of the amount of insoluble red formazan that accumulated in living cells as a consequence of reduction in 2,3,5-triphenyltetrazolium chloride (TTC) with dehydrogenase [76]. Seedling roots were cut off on ice and incubated with 1 mL of 0.4% TTC (Serva; Heidelberg, Germany) for 24 h at room temperature in the dark. Next, the roots were washed with distilled water, transferred to new tubes and flooded with 0.5 mL of 95% ethanol. The samples were homogenized with Tissue Lyser II (Qiagen, Hilden, Germany) and incubated at 55 °C for one hour. After that, another 0.5 mL of 95% ethanol was added to the samples, which were then vortexed and centrifuged for 3 min at 10,000 rcf. Volumes of 800 µL of supernatant and 1400 µL of 95% ethanol were mixed in the spectrophotometer cuvette and the absorbance of the samples was measured at λ = 485. Each experiment was repeated four times.

### 4.5. Lipid Peroxidation Determination

For assessment of lipid peroxidation in seedling roots, the amount of thiobarbituric acid-reactive substances (TBARS) was measured, according to the method described in [77] with some modifications. Whole roots of seedlings (approximately 200 mg) were cut off on ice and homogenized with 3 mL of 10% TCA (Sigma-Aldrich, TO699, St. Louis, MO, USA). Thereafter, the samples were centrifuged (12,000 rpm, 4 °C, 10 min) and 1 mL of supernatant was transferred to glass tubes, filled with 4 mL of 0.5% TBA dissolved in 10% TCA. After incubation at 95 °C for 30 min, the samples were cooled. Next, they were mixed by inversion and centrifuged (5000 rpm, 4 °C, 2 min). The absorbance of the supernatant was measured at 532 nm and corrected for unspecific absorbance at 600 nm (0.5% TBA in 10% TCA was used as a blank). Each experiment was repeated four times and all samples in each experiment were tested in duplicate.

### 4.6. Statistics

Statistical analyses were performed using Statistica 13.3 for Windows (StatSoft, Kraków, Poland). Differences in length and mass of the seedlings, as well as germination rate between treatments were analyse with nonparametric Kruskal–Wallis test. For cell viability and lipid peroxidation quantification, analysis of variance (ANOVA) was used, followed by the Tukey’s post hoc test (if needed). Data are shown as a mean ± standard error (SE).

## 5. Conclusions

To summarize, there is no evidence for the toxic effects of GdVO_4_:Eu^3+^ NPs on vascular plants when used in concentrations of 100 µg/mL and lower. After three days of incubation, GdVO_4_:Eu^3+^ NPs did not affect the number of roots, root length, root mass, hypocotyl length and hypocotyl mass, as well as the germination rate of the wheat seedlings. On the other hand, root cells of the treated seedlings contained less Evans Blue (EB) when compared to the control. The observed decrease in the Evans Blue level indicates that LDNCs affect membrane functioning. The mechanism of that interaction is not known. Thus, it would be interesting to obtain more detailed insights into the effect of these NPs on plants physiology.
